# cdh23 affects congenital hearing loss through regulating purine metabolism

**DOI:** 10.3389/fnmol.2023.1079529

**Published:** 2023-07-27

**Authors:** Shu Yang, Bing-Lin Xie, Xiao-ping Dong, Ling-xiang Wang, Gang-hua Zhu, Tian Wang, Wei-jing Wu, Ruo-sha Lai, Rong Tao, Min-xin Guan, Fang-yi Chen, Dong-hui Tan, Zhong Deng, Hua-ping Xie, Yong Zeng, Zi-an Xiao, Ding-hua Xie

**Affiliations:** ^1^Department of Otorhinolaryngology—Head & Neck Surgery, The Second Xiangya Hospital of Central South University, Changsha, Hunan, China; ^2^Laboratory of Animal Nutrition and Human Health, Hunan International Joint Laboratory of Animal Intestinal Ecology and Health, College of Life Science, Hunan Normal University, Changsha, Hunan, China; ^3^The National & Local Joint Engineering Laboratory of Animal Peptide Drug Development, College of Life Science, Hunan Normal University, Changsha, Hunan, China; ^4^Institute of Genetics, Zhejiang University, Hangzhou, Zhejiang, China; ^5^Department of Human Genetics, Zhejiang University School of Medicine, Zhejiang Provincial Key Laboratory of Genetic & Developmental Disorders, Hangzhou, Zhejiang, China; ^6^Shenzhen Key Laboratory of Smart Healthcare Engineering, Department of Biomedical Engineering, Southern University of Science and Technology, Shenzhen, China; ^7^Department of Otolaryngology—Head and Neck Surgery, The Affiliated Hospital of Xiang Nan College, Chenzhou, China

**Keywords:** congenital hearing loss, Cdh23, gene knockout, ATP, metabolism, regulatory network

## Abstract

**Introduction:**

The pathogenic gene *CDH23* plays a pivotal role in tip links, which is indispensable for mechanoelectrical transduction in the hair cells. However, the underlying molecular mechanism and signal regulatory networks that influence deafness is still largely unknown.

**Methods:**

In this study, a congenital deafness family, whole exome sequencing revealed a new mutation in the pathogenic gene *CDH23*, subsequently; the mutation has been validated using Sanger sequencing method. Then CRISPR/Cas9 technology was employed to knockout zebrafish *cdh23* gene. Startle response experiment was used to compare with wide-type, the response to sound stimulation between wide-type and cdh23^−/−^. To further illustrate the molecular mechanisms underlying congenital deafness, comparative transcriptomic profiling and multiple bioinformatics analyses were performed.

**Results:**

The YO-PRO-1 assay result showed that in *cdh23* deficient embryos, the YO-PRO-1 signal in inner ear and lateral line neuromast hair cells were completely lost. Startle response experiment showed that compared with wide-type, the response to sound stimulation decreased significantly in *cdh23* mutant larvae. Comparative transcriptomic showed that the candidate genes such as *atp1b2b* and *myof* could affect hearing by regulating ATP production and purine metabolism in a synergetic way with *cdh23*. RT-qPCR results further confirmed the transcriptomics results. Further compensatory experiment showed that ATP treated cdh23^−/−^ embryos can partially recover the mutant phenotype.

**Conclusion:**

In conclusion, our study may shed light on deciphering the principal mechanism and provide a potential therapeutic method for congenital hearing loss under the condition of *CDH23* mutation.

## 1. Introduction

Congenital hearing loss is the most common birth defect. In China, it was estimated that 2–3 of every 1,000 newborns suffered congenital hearing loss. The incidence of this disease can reach 0.1–0.3% worldwide (Faundes et al., 2012). It was estimated that 50% of congenital hearing loss is due to genetic causes, and 50% was due to environmental factors such as intrauterine infection, drugs, or trauma (Korver et al., 2017). The genetic causes of congenital deafness can be divided into two categories: syndrome type and non-syndromic type. The former is related to other symptoms and accounts for 30% of cases of genetic causes, while the latter, deafness is the only symptom, accounting for 70% of cases (Smith et al., 2005; Lammens et al., 2013). Congenital deafness can be divided into mild (26–40 dB), moderate (41–55 dB), moderately severe (56–71 dB), severe (71–90 dB), and extremely severe (>91 dB). For patients with congenital hearing loss, there is no cure for hearing loss, and the treatment is limited to hearing aids or cochlear implants (Zhang Z. et al., 2022; Zhang et al., 2023). Many patients cannot adapt to the short-term discomfort caused by it or cannot afford the high price, which leads to treatment interruption.

Congenital deafness can affect unilateral or bilateral hearing. A recent study on hearing loss in children showed that unilateral hearing accounted for 29% and the latter accounted for the remaining 71% (van Beeck Calkoen et al., 2019). Surprisingly, 90% of deaf children have normal hearing parents (Mitchell and Karchmer, 2004). Congenital hearing loss affects children’s language, language acquisition, as well as social and cognitive development. In the past century, more than 180 genes related to deafness have been discovered and studied. The common pathogenic factors in China are *GJB2*, *SLC26A4*, and *CDH23* (Du et al., 2016).

The *CDH23* (*cadherin related 23*) gene is localized on human chromosome 10, which has the function of maintaining intercellular connections and is involved in the formation of static cilia tissue and hair bundles. *CDH23* is an atypical cadherin that forms part of the tip junction and plays a key role in mechano-electrical transduction (MET) of hair cells (Siemens et al., 2004; Sollner et al., 2004). It is expressed mainly on the surface of sensory nerve epithelial cells. *CDH23* is the first gene that has been proven to be related to susceptibility to noise-induced hearing loss (NIHL) in human deafness (Holme and Steel, 2004). Mutations in *CDH23* cause non-syndromic autosomal recessive deafness (DFB12) and Usher Syndrome Type 1D (USH1D) (Bork et al., 2001). *CDH23* plays a crucial role in the normal organization of the static cilia bundle, and mutations in the *CDH23* gene cause disturbances in the stereocilia, leading to disorders in the conversion of sound waves into electrical signals in the hair cells, causing noise deafness and Age-related hearing loss (AHL) (Noben-Trauth et al., 2003).

Exome is widely used in clinical practice at present. Different from genome, exome only focuses on the genome encoding protein (Jelin and Vora, 2018). Exome accounts for just 1.5% of the genome. Exome detection is more economical than genome (Ng et al., 2009). In eukaryotes, precursor mRNA usually includes introns and exons, and different exons are spliced together to form mature mRNA (Wahl et al., 2009). In this way, alternative splicing contributes to the generation of protein subtypes that are structurally and functionally similar but not identical, thus contributing to proteomic diversity (Nilsen and Graveley, 2010). Mutations in exons may lead to changes in protein structure and function. In the inner ear, many important genes have been proven to undergo alternative splicing, and the disorder of this process will greatly lead to hearing loss (Wang et al., 2016; Zhang et al., 2022b). The *CDH23* gene contains 69 exons (Li et al., 2020). At present, the mechanism of inner ear specific alternative splicing of *CDH23* exons remains unclear (Li et al., 2020). Whole exome sequencing can increase the diagnosis of abnormal genes and improve our understanding of the pathogenesis of congenital deafness, which also has the potential to extend known disease phenotypes into the prenatal period. In this study, the zebrafish *cdh23* gene was knocked out and transcriptome data of wild-type zebrafish and *cdh23* knocked-out zebrafish were compared, so as to explore the effect of *cdh23* and its upstream and downstream key genes on congenital deafness and further understand its potential molecular mechanism.

Transcriptomics refers to all RNA transcribed by a cell, tissue, or organism in a particular physiological or pathological state, including coding RNAs and non-coding RNAs (RNAs involved in post-transcriptional control), which will further influence gene expression (Chambers et al., 2019). Organisms regulate metabolism, DNA synthesis and proliferation through transcription (Stark et al., 2019). The transcriptome provides us with a comprehensive understanding of molecular genetic information, which directly affects the proteome, and thus indirectly enables us to have an adequate understanding of biological physiological or pathological processes (Song et al., 2019). Transcriptomics has the characteristics of high throughput, low cost, fast and accuracy compared with other omics. There are few transcriptome studies on congenital deafness in zebrafish.

In the past few years, zebrafish (Danio rerio) has been a popular animal model in hearing research (Yang et al., 2017). As an ideal animal model for hearing research, zebrafish has the following advantages: First, it has hair cells in the inner ear, allowing researchers to easily access the hair cells. Secondly, within a few days after *in vitro* fertilization, zebrafish can develop rapidly and their hair cells mature within a day (Kindt et al., 2012; Yang et al., 2017). Third, gene expression of zebrafish fluorescent structure is easy to achieve. In addition, the transparent embryos and larvae make it a good live imaging model (Kindt and Sheets, 2018; Pickett and Raible, 2019). Therefore, we performed transcriptome sequencing on *cdh23* mutant zebrafish in this study. mRNA expression data of wild-type (WT) and *cdh23*^−/−^ were compared. By studying the differences in gene expression levels and combining them with various bioinformatics analysis methods, we attempted to decipher the potential molecular mechanism of this gene and its upstream and downstream key genes on hearing in zebrafish from a molecular perspective. At the same time, comparative transcriptome analysis results showed that the gene expression level changed significantly after the *cdh23* gene was knocked out in zebrafish. By analyzing these differentially expressed genes with the help of bioinformatics methods, we had an in-depth understanding of the mechanism of the *cdh23* gene on zebrafish hearing.

The hair cell bundle transduced the mechanical energy into electrical energy by gating ion channels, MET and synaptic transmission of the hair cells are energy-demanding processes, relying on ATP through oxidative phosphorylation to meet these high metabolic demands (Holmgren and Sheets, 2021; Zhang et al., 2022a). ATP can activate ionotropic purinergic (P2X7) to influence cell functions. Previously studies have shown that ATP depletion led to histone deacetylation and eventually resulted in hair cell death (Fan et al., 2020), ATP also plays an important signaling molecule in the inner ear (Housley, 1998). Overall, our finding demonstrated that loss of *cdh23* resulted in defective purine metabolism, and consequently, insufficiency of ATP, which is of great importance for the normal function of hair cells.

## 2. Materials and methods

### 2.1. Subjects information

#### 2.1.1. Families with congenital deafness

**Figure 1:**
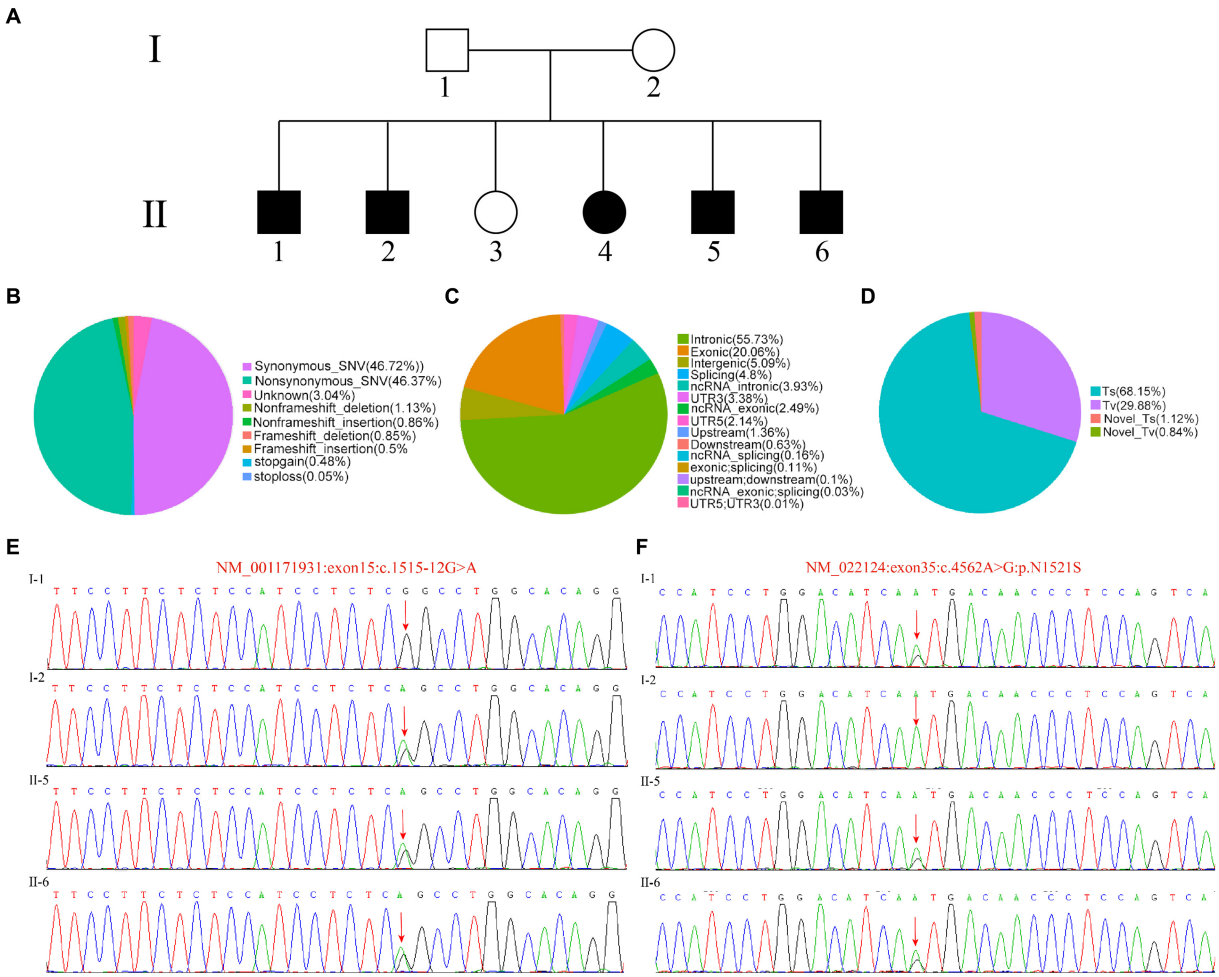
Identification of patient mutation. **(A)** Patient information and clinical diagnosis. **(B)** The distribution ratio of SNV/InDel sites in different regions of the whole genome. **(C)** The distribution ratio of SNV/InDel sites of different functional types. **(D)** The distribution ratio of SNV types of conversion/transversion. **(E,F)** Sanger sequence of the target sequence.

The proband and his family members were recruited from the otolaryngology Department of The Second Xiangya Hospital. A complete medical examination history was performed, including otolaryngology status, pure tone audiometry, and brainstem evoked response audiometry for the patient with hearing impairment. The specific information is shown in Figure 1A. Informed consent was obtained and the study was approved by the ethics committee of The Second Xiangya Hospital of Central South University.

#### 2.1.2. Whole exome sequencing

Genomic DNA was extracted from peripheral blood samples and DNA concentrations were detected by a NanoDrop 2000 (Thermo Fisher Scientific, MA, United States). Sample integrity and purity were detected by agarose gel electrophoresis. The exonic DNA fragments were subjected to end-repair, 3′ adenylation, and adaptor ligation. Following adaptor ligation, the DNA library was amplified according to standard Illumina protocols. Exome capture was then performed using the SureSelectXT Reagent kit (Agilent Technologies, CA, United States) and SureSelectXT Human All Exon Kit V6 (Agilent Technologies, CA, United States). Then the library was qualified by an Agilent 2,100 bioanalyzer (Agilent Technologies, CA, United States). The qualified libraries were sequenced on the Illumina Hiseq platform with 2 × 150 bp double-terminal sequencing mode.

The human reference genome assembly hg19 was used as a reference sequence in this study. Then BWA’s MEM algorithm was used to compare the raw sequence data of each sample with the reference genome and obtain the preliminary comparison result in BAM format. Then the comparison results of each sample including the number and proportion of the sequence on the comparison and the average coverage depth, etc. are counted using Picard software. Finally, the GATK HaplotypeCaller method was used to detect the SNV/InDel of each sample. These SNV/InDels were annotated with ANOVA to quickly find the most biologically significant SNV/InDel. To further verify the candidate gene *CDH23*, we extracted the genomic DNA, using PCR to amplify the target region, after purification, we sent the PCR product for sequencing.

### 2.2. Establishment of *cdh23*-deficient zebrafish lines and analyze the role of cdh23 in auditory

#### 2.2.1. Ethics statement

All animal experimental procedures were in compliance with local and international regulations; animal experiments were ethically approved by the institutional animal care committee of Experimental Animals of Hunan Normal University, Hunan, China.

#### 2.2.2. Zebrafish maintenance and manipulation

Tübingen (TU) zebrafish (Laboratory of Animal Nutrition and Human Health, College of Life Science, Hunan Normal University) was used in this study. Zebrafish embryos were obtained through natural mating and maintained at 28.5°C in the fish facility. The embryo was treated with 0.2 mM 1-phenyl-2-thiourea (PTU) to inhibit pigment development. When developed to desired stages, embryos were collected and fixed with 4% paraformaldehyde (PFA) in phosphate buffered saline (PBS) overnight at 4°C.

#### 2.2.3. Zebrafish *cdh23* knockout by CRISPR/Cas9

**Figure 2:**
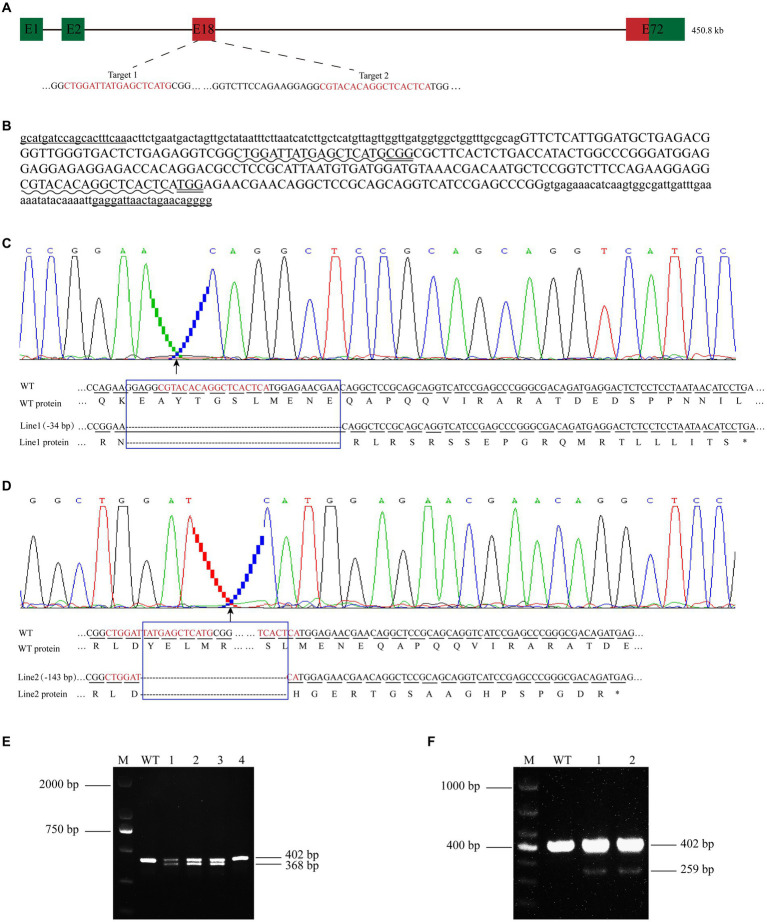
Establishment of *cdh23* gene knockout lines. **(A)** The schematic diagram of the *cdh23* gene target site. The gene is 450.8 kb in length and has 72 exons. Exon 18 is the target for CRISPR/Cas9 gene editing in zebrafish *cdh23*. **(B)** Design of *cdh23* gene knockout target, the uppercase letter is the exon, the lowercase letter is the intron, “___” is the detection primer, “ ~ ~ ~ ~” is the target site sequence, and “=====” is the PAM sequence. The distance of the two target sites is 115 bp; **(C,D)** Partial DNA and the corresponding amino acid sequence of *cdh23* line 1 and line 2 mutant, respectively. The blue box indicates the missing sequence. Line 1 has a total deletion of 34 bp, and protein translation is terminated prematurely. Line 2 missed 143 bp, and protein translation also terminated prematurely. **(E,F)** DNA agarose gel of *cdh23* line 1 and line 2, respectively.

The *cdh23* mutant lines were generated with the CRISPR/Cas9 system following previous methods with a slight modification. At first, the complete gene sequence and amino acid sequence of zebrafish *cdh23* were accessed from NCBI^1^. The optimal target sites were screened from the website^2^ and finally were selected on the second exon of the *cdh23* gene (Figures 2A,B). T7 promoter sequence and protection base were added to the 5′ terminus of the sgRNA forward primer for sgRNA synthesis *in vitro*. The forward primer (sgRNA-F) and the reverse primer (sgRNA-R) were used for PCR amplification. Finally, the detection primers CDH23-F and CDH23-R were designed by Primer 3.0 software. The specific primers and template sequences are shown in Table 1. The sgRNAs were *in vitro* synthesized using T7 transcription kit (Thermo Fisher Scientific). sgRNA1 and sgRNA2 were purified and recovered using RNA purification kit (Qiagen). Cas9 protein was purchased from Thermo Fisher Scientific. Then, each one-cell stage embryo was injected with a 1-nL of the solution containing ~50 ng/μL of sgRNA1, ~50 ng/μL sgRNA2, and 1 μg/μL Cas9 proteins unless otherwise indicated. After injection, the embryos were incubated at 28.5°C.

**Table 1:** Primers and template sequences involved in this paper.

Primer	Sequences (5′→3′)
Template DNA	TTTTAGAGCTAGAAATAGCAAGTTAAAATAAGGCTAGTCCGTTATCAACTTGAAAAAGTGGCACCGAGTCGGTGCTTT
sgRNA1-F	GCGTAATACGACTCACTATAGGCTGGATTATGAGCTCATGGTTTTAGAGCTAGAAATAG
sgRNA2-F	GCGTAATACGACTCACTATAGGCGTACACAGGCTCACTCAGTTTTAGAGCTAGAAATAG
sgRNA-R	AAGCACCGACTCGGTGCCACT
CDH23-F	GCATGATCCAGCACTTTCAA
CDH23-R	CCCCTGTTCTAGTTAATCCTC

When the embryos developed to 36 hpf, the control embryos and injected embryos were randomly selected for effectiveness testing. As the two target sites are 115 bp away from each other, if both target sites work well, there would be a ~115 bp deletion, compared with control. Once the mutation was confirmed in injected embryos, the remaining ones were raised to 45 dpf, each fish was followed by fin clip, genomic DNA extraction and genotyping, the ones with a ~115 bp deletion are raised to adulthood as founders (F0), then crossed with WT (namely 402 bp TU wild-type bands). F1 generation mutant (bands less than 402 bp) was obtained. The F1 mutants were also performed genotyping, and the gene knockout bands that smaller than 402 bp were recovered by gel cutting and sequencing. We chose the mutant lines that cause frameshift of protein coding, and the stable genetic *cdh23* gene knockout mutants of zebrafish were obtained.

#### 2.2.4. Hair cell staining of zebrafish

Ten gene knockout and control zebrafish larvae in indicated stages were placed into a 24-well plate with 2 μM YO-PRO-1, respectively, and incubated overnight in dark. The next day, the YO-PRO-1 solution was washed by egg water, 3 times, 5 min interval. Then the zebrafish were anesthetized in 0.4% MS-222 (tricaine) solution, 3% methylcellulose was added to the concave slide, and then the zebrafish was embedded in methylcellulose. The hair cells were observed under a fluorescence microscope, photographed and counted.

#### 2.2.5. Startle response of zebrafish larvae

The Startle Response was carried out as previously described (Wang et al., 2017). First, about 15 of 5 dpf zebrafish larvae were raised in a Petri dish with a 2 mm thin layer of egg water. Tone bursts of 400 Hz at different sound intensities were delivered to the Petri dish through a mini vibrator (QY50R Z). Using a digital camera (Basler acA1300–200 μm) to record the movement of each larva at 120 frames per second (fps) and analyzed by a customized software developed in MATLAB (MathWorks, MA, United States). The distance of larvae’s C-shape movement upon sound stimulation was used as a measure of its auditory startle response.

#### 2.2.6. RNA-seq analysis

Total RNA was extracted using the mirVana miRNA Isolation Kit (Ambion) and the Agilent 2,100 Bioanalyzer (Agilent Technologies, Santa Clara, CA, United States) was used to evaluate RNA integrity. The samples with RNA Integrity Number (RIN) ≥ 7 were subjected to the subsequent analysis. According to manufacturer instructions, TruSeq Stranded mRNA LTSample Prep Kit (Illumina, San Diego, CA, United States) was used to construct libraries. Then these libraries were sequenced on the Illumina sequencing platform (HiSeqTM 2,500 or Illumina HiSeq X Ten) to generate 125 bp/150 bp paired-end reads. After the raw data was processed by Trimmomatic, the cleaned reads were mapped into the GRCz11 reference genome using Hisat2. FPKM value of each transcript was calculated using Ballgown. The FPKM of each transcript is calculated by using Cufflinks. The differentially expressed transcripts were filtered with value of *p* <0.05 and | log_2_ratio| >1 as thresholds.

#### 2.2.7. Multiple bioinformatics analysis of quantitative transcriptomic data

DAVID(Database for Annotation, Visualization and Integrated Discovery)comprises a comprehensive knowledge base. It provides a set of gene functional annotation and enrichment tools for the investigator to reveal the biological content captured by high throughput data (Huang et al., 2009a,b). Reactome is an open access pathway database that enables researchers to find, organize and utilize biological information to support data integration, analysis, and visualization (Fabregat et al., 2018; Jassal et al., 2020). In this study, all DEGs were imported into Reactome for pathway analysis. *p* < 0.05 was set as the statistical cut-off line for the identification of significant pathways. The STRING (Search Tool for the Retrieval of Interacting Genes/Proteins) is a database of known and predicted protein–protein interactions (Szklarczyk et al., 2017, 2019). In order to understand the relationship between genes, we imported the list of differential genes into the STRING database, and the results generated were used for network construction based on the Cytoscape platform.

### 2.3. Ethics statement

All embryos were handled according to relevant national and international guidelines ‘Act on Welfare and Management of Animals’. Full details of the study were approved by Ethics Committee of The Second Xiangya Hospital of Central South University and Ethics Committee of Hunan Normal University.

## 3. Results

### 3.1. Whole-exome sequencing and sanger sequence validation

The proband II-5, patient II-6 and the parents’ samples from the family were successfully sequenced and variants were detected. We not only classified the position of SNV/Indel locus relative to the genome and the functional type of SNV/Indel, but counted the number and proportion of SNVs of conversion/transversion types (Figures 1B–D). Compared with other mutation types, we can see that intron mutations were the most, about 34%, followed by exon mutations (Figure 1B). As we all known, the mutations that occurred in the exon region were likely to directly lead to changes in the final product, leading to traits changes. While Figure 1C showed the distribution ratio of SNV/InDel sites of different functional types, the SNV mainly includes synonymous and missense or missense mutations (nonsynonymous). The InDel type including frameshift insertion and deletion, nonframeshift insertion and deletion were relatively small. The SNV distribution proportion of conversion/transposition types were shown in Figure 1D, in which the Ts accounts for 68.15% and the Tv for 29.88%.

Then, all SNV/InDel sites were filtered according to the following criteria, and the sites meeting these conditions were reserved for further analysis: First Priority is First, and First1 is conservative; Preferentially select low frequency including the frequency of 1,000 Genomes was lower than 0.01 (dominant genetic model) or 0.05 (recessive genetic model); SNP calling quality and genotyping with high quality are preferentially selected; SIFT Score Pred, PolyPhen V2 Score Pred and Mutation Taster Pred were ranked D. The results showed that I-1 contains NM_022124:exon35:c.4562 A > G:p.N1521S mutation site and I-2 with NM_001171931:exon:c.1515-12G > A mutation site, the proband II-5 and patient II-6 carries both NM_001171931:exon:c.1515-12G > A and NM_022124:exon35:c.4562 A > G:p.N1521S sites, to further validate the WES results, the genomic DNA of the parents and patients II-5, II-6 were extracted, PCR was performed, Sanger Sequencing confirmed the WES results (Figures 1E,F). Based on the above conditions, we selected *CDH23* as a candidate gene.

### 3.2. Establishment of zebrafish *cdh23* knockout lines

To investigate the function of *cdh23*, we generated *cdh23* mutant zebrafish using CRISPR/Cas9 system. Two independent mutant alleles were obtained from different F0 founders, genotyping result showed that, in F0-1 offsprings, there is a small band below the wild-type band (Figure 2E). F0-2 offsprings also had a small band (Figure 2F), proving that the mutation can be inherited to the next generation. The two knockout bands were purified and recovered, then sent for DNA sequencing and blast with wild type, compared with the wild type, F0-1 offsprings have a 34 bp deletion (Figure 2C), while F0-2 offsprings lost 143 bp (Figure 2D), the rest of the embryos are raised to adult and the *cdh23* mutant line was established as line1, and line2, respectively. Both the two mutants led to frame-shift mutations of the open reading frame, and premature stop codons that can abolish all functions of *cdh23* (Figures 2C,D). It indicates that *cdh23* gene knockout lines were successfully established.

### 3.3. Effects of *cdh23* gene on zebrafish morphology

F1 generation zebrafish of line1 and line2 were incrossed to obtain F2 generation zebrafish, and embryonic morphological changes were observed. Before the zebrafish developed to 72 hpf, there was no obvious defect. After 96 hpf, compared to sibling (Supplementary Figure S1A), about 25% of the embryos in line1 zebrafish had a slight curve in the tail (Supplementary Figure S1B). At 7dpf, the embryos with curved tails are dead. cdh23 line2 mutant embryos showed the same phenotype (data not shown). To further check if *cdh23* deficient leads to embryonic lethality, we collect the dead embryos and genotyped them, and the results showed that all the embryos with serious malformations that led to death were *cdh23*^−/−^ (Supplementary Figure S1C). These results showed that the loss of *cdh23* gene resulted in embryonic lethality.

### 3.4. Effects of *cdh23* on the hair cells of lateral line nerve of zebrafish

**Figure 3:**
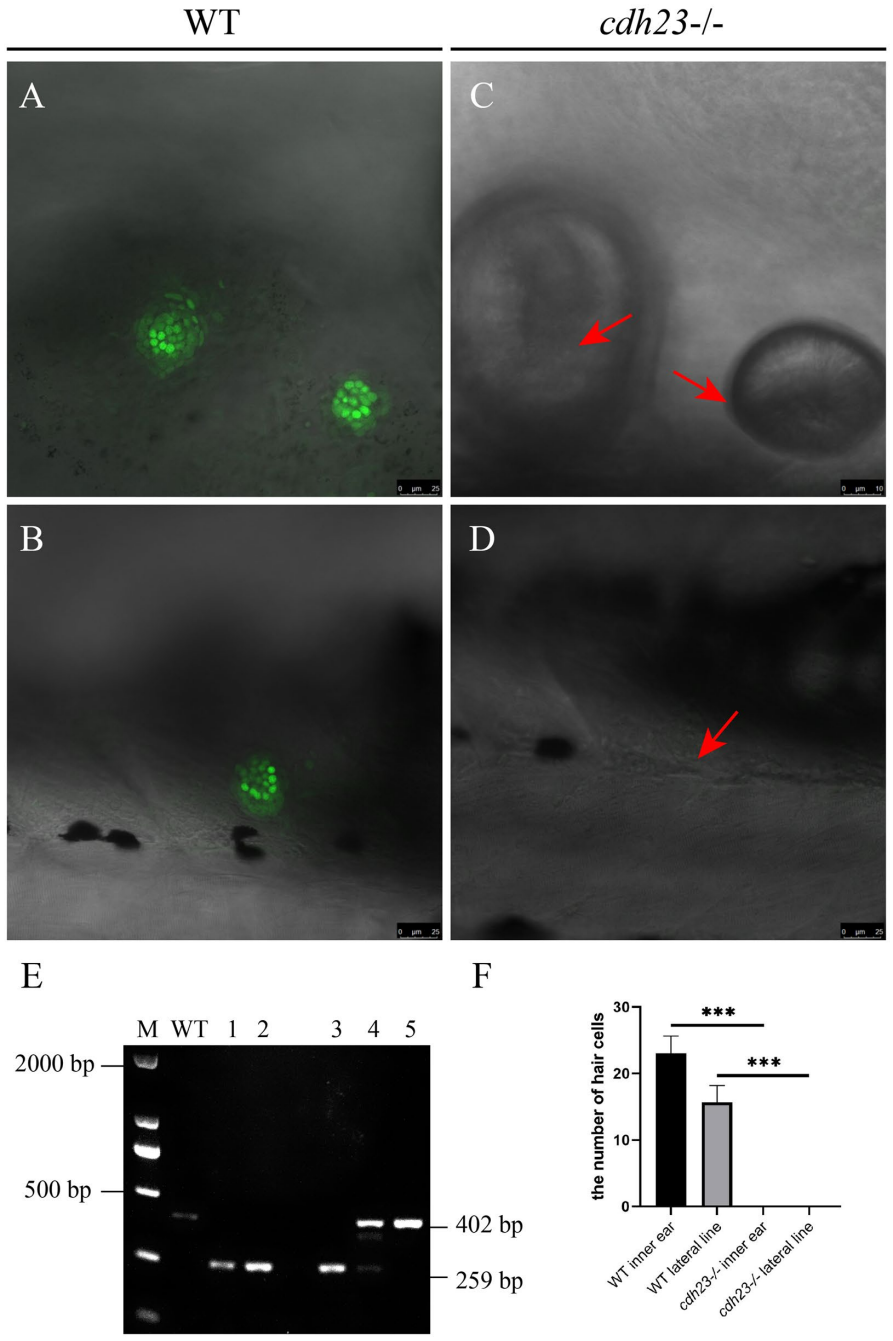
Knock out of *cdh23* resulted in impaired hair cell function. The function of hair cells is evaluated by performing a YO-PRO-1 uptake experiment at 6 dpf. The ear hair cell **(A)** and lateral neuromast **(B)** uptake YO-PRO-1, while in the *cdh23* homozygous mutant, the ear hair cell **(C)** and lateral neuromast **(D)** (arrow) do not have YO-PRO-1 fluorescence signal. **(E)** Genotyping results confirmed that the embryos with impaired function of hair cells are homozygous mutants. **(F)** The numbers of hair cells in each group, ***, significant difference, *p*<0.001.

The previous study showed that *CDH23* is expressed at the tip of hair cells (Sollner et al., 2004). Hair cells are highly energy demanded cells, and inefficient of ATP can result in impaired functions; P2RX7 is an ATP gated ion channel, which can uptake YO-PRO-1 through endocytosis. To further confirm the mutant phenotype, YO-PRO-1 staining was performed to assess the function of hair cells (Vargo et al., 2017). At 3 dfp, the hair cells in the otic vesicle and lateral line of control embryos can uptake YO-PRO-1, while about 25% of *cdh23* inbred embryos without any fluorescence signal (Figures 3A–D). The embryos without fluorescence signal were collected and genotyping results confirmed that the embryos lost fluorescence are homozygous mutants (Figure 3E). These results indicated that the deletion of *cdh23* could impair the function of ion channel P2RX7.

### 3.5. Startle response experiment

**Figure 4:**
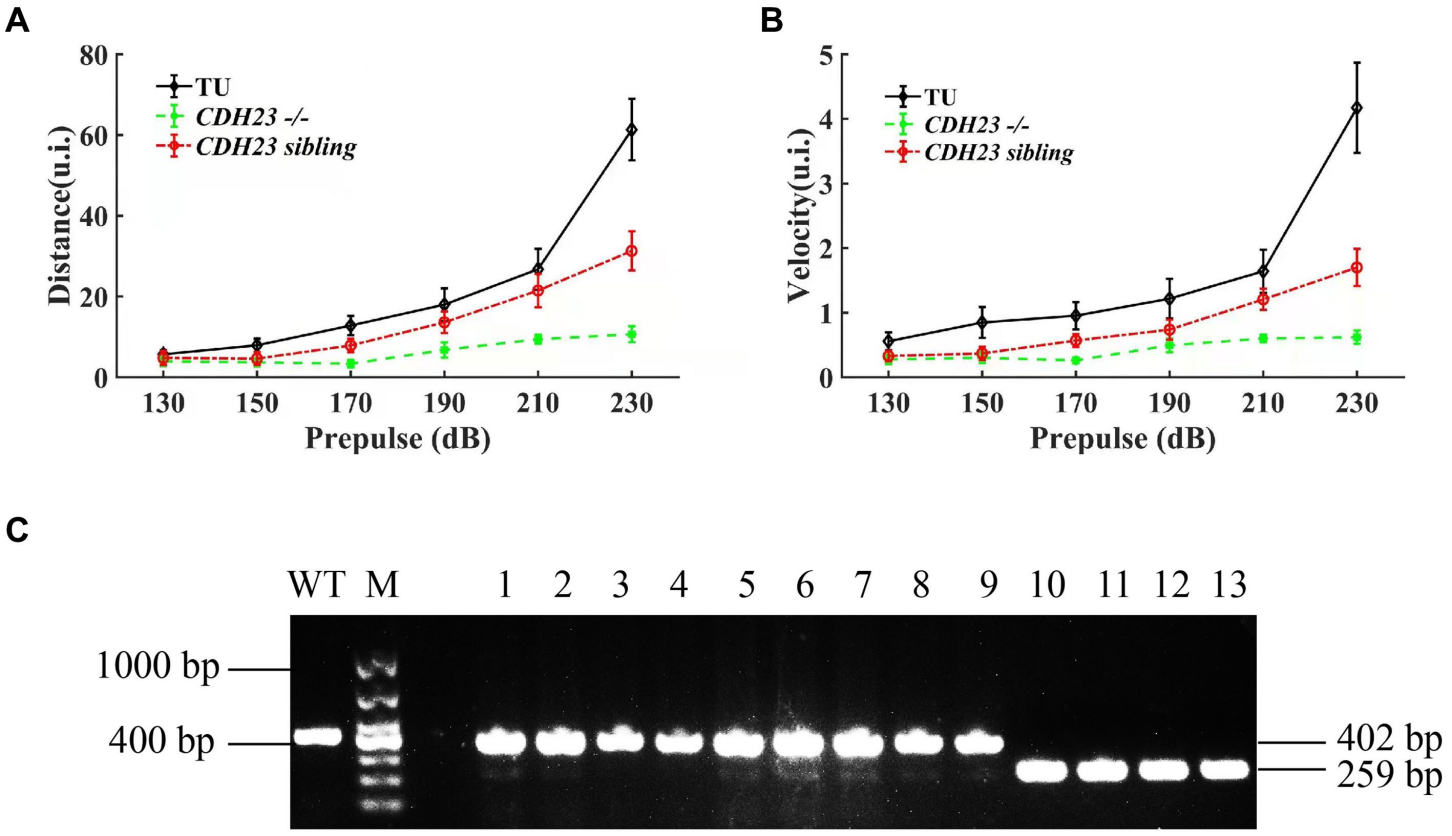
The results of startle response for zebrafish juveniles. **(A)** Under the action of prepulse of different intensities, the movement distance of *cdh23* homozygous mutants and heterozygous juveniles was significantly lower than that of the wild type at the same time. **(B)** Under the stimulation of prepulse of different intensities, in *cdh23* homozygous mutants and sibling juveniles, the movement speed of sibling juvenile fish is significantly lower than that of wild type. All zebrafish juveniles used developed to 6 dpf. **(C)** Genotyping results of zebrafish juveniles after startle response.

YO-PRO-1 staining result showed that in *cdh23* mutant embryos, the hair cells have no fluorescence, which is similar to the previously reported (Wang et al., 2017). In order to check if *cdh23* indeed affects hearing, we performed a startle response experiment as previously described, we incrossed the *cdh23*^+/−^ fish. We recorded the response by zebrafish larvae’s moving distance upon sound stimulation individually at 6 dpf. Next, we performed a genotype experiment, and then analyzed the wide-type, heterozygous group and homozygous mutant after startle response (Figure 4C). Our results showed that there is no difference among them in 130 dB prepulse. The average distance in the wide-type group increased from 5.706 mm to 61.335 mm (Figure 4A), the average velocity increased from 0.137 mm to 0.698 mm (Figure 4B), with the increase of prepulse, which means that the wide-type fish response quick to the prepulse increase. While the average distance in *cdh23* homozygous mutant group increased from 3.975 mm to 10.674 mm, the average velocity increased from 0.074 mm/s to 0.103 mm/s, almost do not affect by the sound stimulus. The average distance of the heterozygous mutant group is between wide-type and homozygous mutant groups, indicating that the response of the sound stimulus is also defective in heterozygous embryos.

### 3.6. RNA-seq analysis of zebrafish with the knockout of *cdh23* gene

#### 3.6.1. Transcriptomics profiles of zebrafish with *cdh23*^−/−^

**Figure 5:**
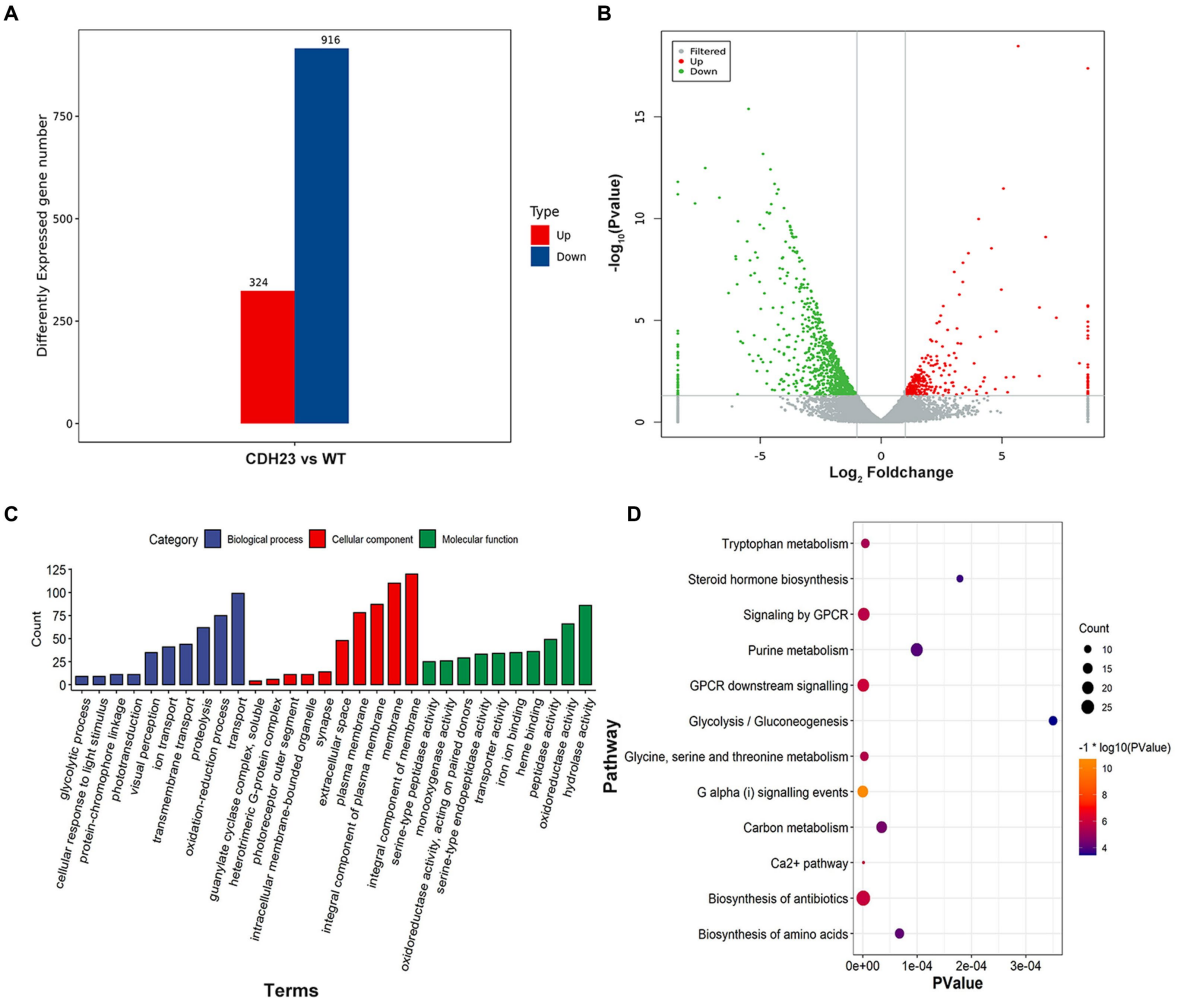
Transcriptomics landscape with *cdh23*^−/−^. **(A)** 1240 genes were identified as DEGs (*p* value < 0.05 and ∣log_2_FC∣ > 1) with 324 being up-regulated and 916 being down-regulated. **(B)** Gene distribution according to expression level between case and control; **(C,D)** The important terms and pathways are closely related to phenotypic characteristics based on DEGs identified in this study.

To identify those differentially expressed genes (DEGs), the Fragments per Kilobase Million (FPKM) of each transcript from the control and case were compared. The student’s test was used to filter DEGs with the threshold of **|**log_2_ratio**|** > 1 and value of *p* <0.05. A total of 1,240 genes were identified as DEGs between case and control subjects (Figure 5A). Among them, 324 genes were up-regulated and 916 were down-regulated (Figure 5B). Gene functional annotation analysis demonstrated that DEGs were significantly enriched in biological processes including “transport,” “ion transport,” “transmembrane transport” and “glycolytic process.” In the cellular component (CC) category, DEGs were mainly involved in “membrane,” “plasma membrane” and “integral component of membrane.” In the molecular function (MF) category, they were mainly enriched in “oxidoreductase activity,” “iron ion binding” and “transporter activity” (Figure 5C). Most of the terms were related to ion transport and membrane potential conversion. Specifically, DEGs such as *atp1b2b*, *clic5b*, and *myof* play significant roles in the above terms.

Notably, pathway analysis demonstrated that a large proportion of DEGs including *pklr, gck*, and *eno2* were enriched in metabolic pathways including “glycolysis/ gluconeogenesis,” “glycine, serine and threonine metabolism,” “Carbon metabolism,” “Ca^2+^ pathway” and “purine metabolism” (Figure 5D). The results indicated that energy production and directional supply were disturbed under the condition of *cdh*23 deficiency. It could lead to the metabolic disorder of ATP and further regulate sensory signaling in a cooperative way with the purine signal transduction system.

#### 3.6.2. PPI analysis and network construction and visualization

**Figure 6:**
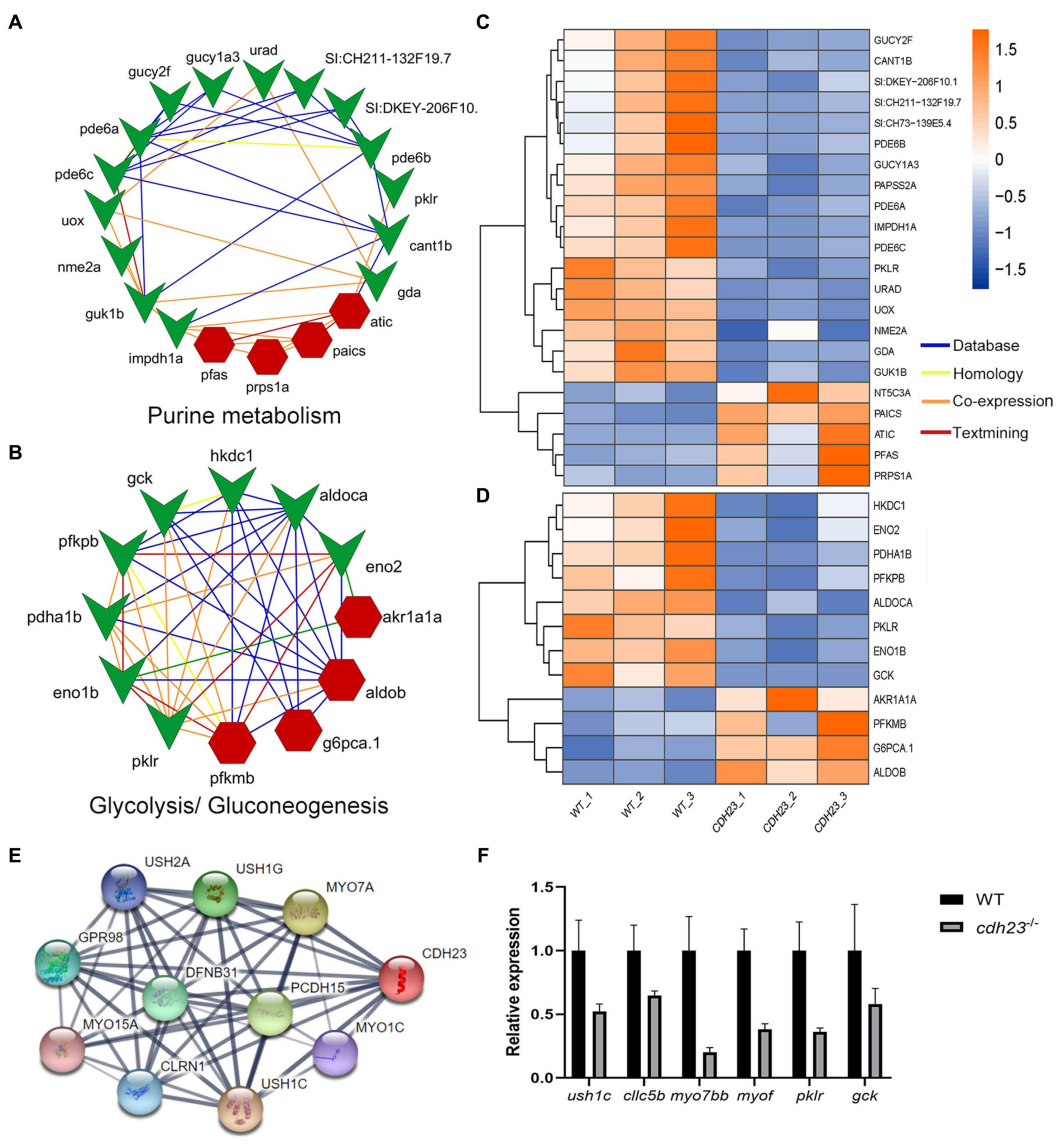
Network construction and visualization based on specific pathway and gene. **(A,B)** Key genes involved in glycolysis/gluconeogenesis and purine metabolism and their interactions; **(C,D)** The expression heatmap of these genes between the control and the *cdh23*^−/−^ group. **(E)** Genes closely related to *cdh23* in the string database. **(F)** RT-qPCR analysis evaluating the expression change of genes related to *cdh23*.

To further understand the molecular mechanism of *cdh23*^−/−^ resulted in deafness from a systematic view, sub regulatory networks were constructed around *cdh23* (Figure 6). DEGs enriched in causal pathways were used for network construction and visualization. The results showed that the co-expression genes such as *nme2a*, *pklr*, and *gck* play crucial roles in purine metabolism and glycolysis/gluconeogenesis pathways in the *cdh23*^−/−^ (Figure 6). It indicated that the loss of *cdh23* will lead to a series of peripheral reactions, meanwhile; all the responsive genes may form a complex network and regulate the production and metabolism of ATP, further mediating the binding process of neurotransmitter analog and P2RX7 receptor on the surface of hair cells.

### 3.7. ATP compensation partially restored *cdh23* deficient

**Figure 7:**
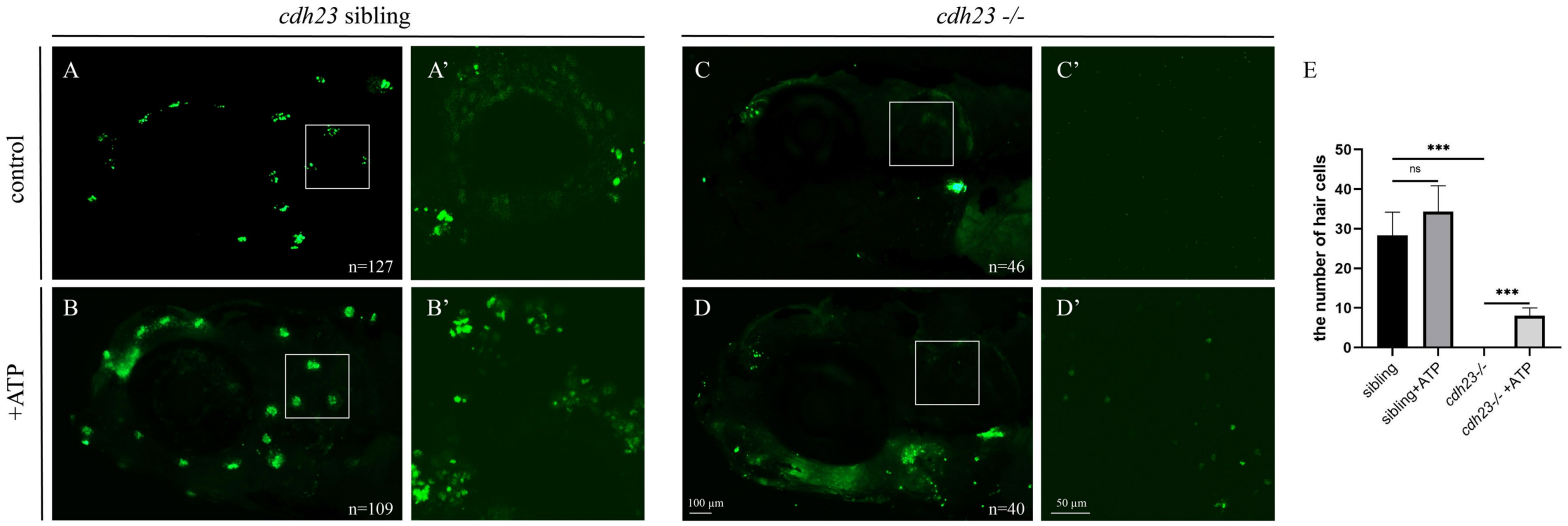
ATP compensation partially recovered the *cdh23* mutant phenotype. **(A)** The hair cells in ear and around the eye can uptake YO-PRO-1 dye while the *cdh23* mutant embryos cannot **(C)**, **(A’)**: the hair cells in the control inner ear, **(C’)**: the hair cells in *cdh23* mutant embryos. The ATP supply of control **(B)**, hair cells were specifically stained by YO-PRO-1 dye, in *cdh23* mutant **(D)**, after ATP adjunction, the function of hair cells in inner ear were partially recovered, while the hair cells around the eye still impaired. **(A’–D’)** is the otic regions of **(A–D)**, respectively. **(E)** The numbers of hair cells in each group, ns, no significant difference, ***, significant difference, *p*<0.001.

Transcriptome and qPCR results indicated that loss of *cdh23* resulted in impaired metabolism and insufficient ATP. As P2RX7 is an ATP-gated ion channel, we next determined whether the shortage of ATP caused YO-PRO-1 cannot enter into hair cells. We performed ATP compensation experiment in sibling embryos after the YO-PRO-1 assay as previously described (Figure 7A). We can see that the hair cell in the inner ear and lateral line cells can be stained by YO-PRO-1 (Figures 7A,A'), while the signal in *cdh23*^−/−^ embryos is completely lost (Figures 7C,C'). Next, we added 3 μM of ATP solution in E3 water for 6 h, the result showed that the hair cells still have fluorescence signal in the sibling group (Figures 7B,B'), while in the *cdh23*^−/−^ group, we can see the YO-PRO-1 signal was partially restored in the inner ear and lateral line hair cells (Figures 7D,D'). These results suggest that the knockout of *cdh23* resulted in impaired metabolism caused by the shortage of ATP and ATP application can partially recover the function of hair cells.

## 4. Discussion

Sound signals are acquired and digitized in the cochlea by the hair cells that further transmit the coded information to the central auditory pathways. Any defect in hair cell may induce the auditory system problems and affect hearing-based brain functions (Liu et al., 2021).

Previous study showed that pathogenic *CDH23* are variants responsible for both recessive DFNB12 nonsyndromic hearing loss (NSHL) and Usher syndrome 1D (USH1D) (Ramzan et al., 2020). In zebrafish *cdh23*^−/−^ embryos, the auditory hair cells were impaired, which resulted in hearing loss, however, the downstream signal and regulate network is still largely unknown. In this study, we knocked out zebrafish *cdh23* to explore the physiological and morphological functions of this gene. Compared the transcriptomes of zebrafish between *cdh23* gene knockout and wild-type zebrafish, we found that the differential genes were significantly enriched in pathways related to ion transport or metabolism. For examples: “ion transport,” “synapse organization,” “calcium ion transmembrane transport,” “actin filament binding,” “voltage-gated ion channel activity” and “calcium channel activity.” The genes involved in these pathways: *atp1b2b*, *cacna1fa*, *clic5b*, *myof*, etc.

The normal growth and development of organisms need to maintain the relative balance of ions in cells, so ion transport plays an important role in maintaining normal pH and ion concentration stability (Foulke-Abel et al., 2016; Seifter, 2019; Guo et al., 2021). Calcium ion transmembrane transport Calcium pump (A.K.A.CA2 + -ATPase or SERCA) is a membrane transport protein that is ubiquitous in the endoplasmic reticulum (ER) of all eukaryotic cells. As a calcium transporter, it maintains a low cytosolic calcium level, allowing a large number of signal transduction pathways and physiological processes (such as synaptic transmission, muscle contraction, and fertilization) to cope with the physiological mechanisms produced by the body in different states (Primeau et al., 2018). The transmission of sound signals depends on neurons, and synaptic organization and synaptic transmission are important connections between neurons, which play an important role in signal transmission. The analysis of the transcriptome data in this study shows that the down-regulated genes such as *atp1b2b*, *clic5b*, and *cacna1fa* are involved in metabolism, resulting in insufficient ATP, as hair cells have high energy demands and contribute to intracellular calcium homeostasis (Holmgren and Sheets, 2021), so as to affect the normal functions of hair cells and downstream signal transduction. Therefore, we speculate that the expression of these down-regulated genes may further prohibit signal transduction; thereby making hearing function blocked.

**Figure 8:**
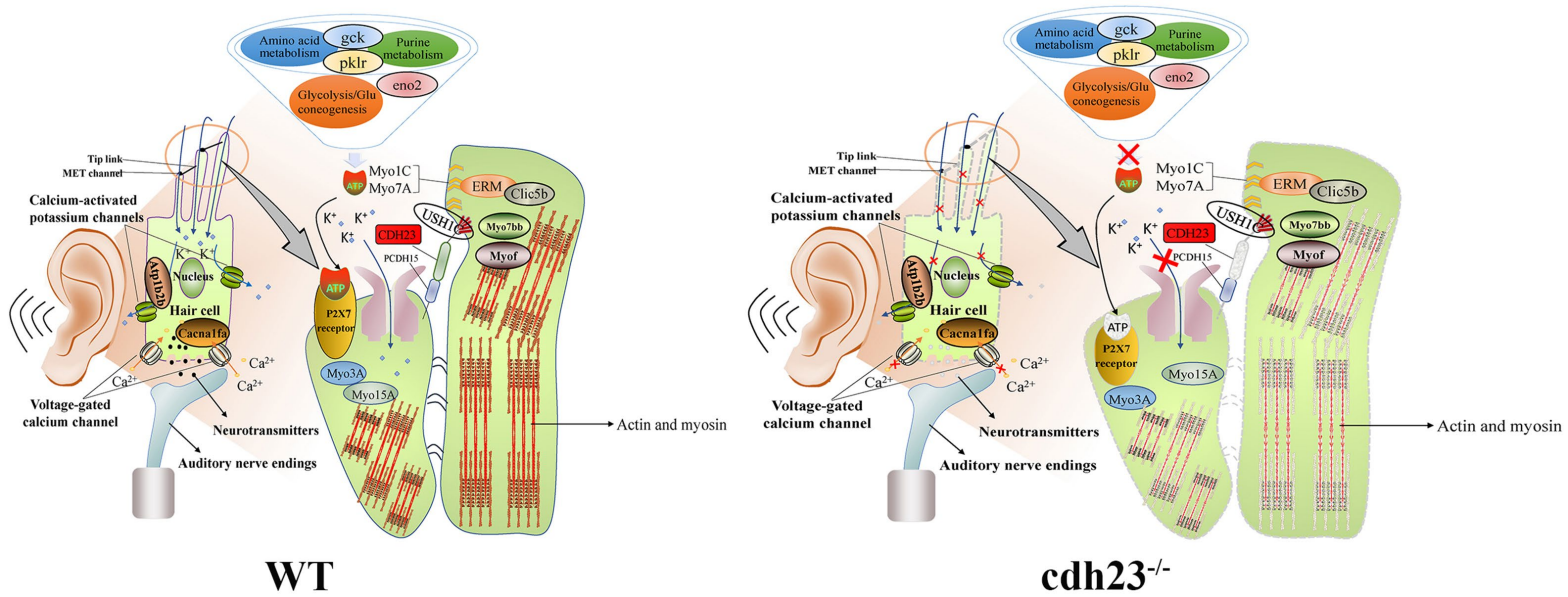
Schematic diagram of the potential molecular mechanism regarding *cdh23* on congenital hearing loss. In WT, candidate genes (*clic5b*, *myof*, *myo7bb*) may be associated with the cytoskeleton of actin and participate in the movement of cilia in inner ear hair cells. Purine and amino acid metabolism yield sufficient ATP, and when ATP binds to the P2X7 receptor, these genes (*atp1b2b*, *cacna1fa*) may mediate the opening of related ion channels, thereby completing the acoustic-electric conversion. While in *cdh23*^−/−^ embryos, purine metabolism is disrupted, resulting in a shortage of ATP, leading to dysfunction of the ion channel P2X7 receptor, and hence interrupting the acoustic-electric conversion, resulting in congenital hearing loss.

Actin is a conserved cytoskeletal protein with essential functions. As the molecular motor of the cytoskeleton, myosin is a multifunctional protein whose main function is to provide the necessary force for muscle contraction, cytoplasmic flow, organelle movement, material transportation, mitosis, cytokinesis, and cell top growth (Lepelletier et al., 2006; Carisey et al., 2018). In this study, differential genes (*myof*, *myo7bb*) were significantly enriched in actin filament binding, three-dimensional organization of inner ear receptor cells and other pathways (Figure 8). The conduction of sound requires an electromechanical conversion mechanism, and the electromechanical conversion mechanism requires ion transport inside and outside the cell and the deflection of the static cilia (Korver et al., 2017; He et al., 2021). The deflection of the static cilia will cause the contraction or relaxation of actin, which further causes the morphology of the cilia to change, which may cause the signal, to be passed on.

MYOF is a phospholipid-binding protein that relies on calcium ions to play a biological role. It has multiple C2 domains, promotes cell fusion, endocytosis, participates in membrane repair, vesicle transport, and muscle regeneration and development and other cell biological functions. The dysfunction of this protein is related to muscle dysfunction. However, little is known about MYOF in deafness research. More and more studies have identified MYOF as an oncogenic protein. It is overexpressed in a variety of human cancers and promotes tumorigenesis, tumor cell movement, proliferation, migration, epithelial to mesenchymal transition, angiogenesis, and metastasis (Dong et al., 2019).

ATP1B2B encodes a part of ATPase, a non-catalytic component of the active enzyme, which catalyzes the hydrolysis of ATP and combines with the exchange of Na (+) and K (+) ions on the plasma membrane. Due to changes in the expression levels of enzymes that regulate ATP synthesis, the production of ATP is blocked so that it cannot provide energy for downstream acoustic and electrical transduction signaling. *atp1b2b* gene may have heart failure; reduce the number of sensory hair cells in the cochlea and neuro transformation, and the role of abnormal sensory responses (Guo et al., 2021). *clic5b* is required for normal hearing (Seco et al., 2015). It is necessary for the formation of stereocilia in the inner ear and normal development of the organ of Corti and can insert into membranes and form poorly selective ion channels that may also transport chloride ions(Berryman et al., 2004). On the other hand, *CLIC5B* plays a role in the regulation of transepithelial ion absorption and secretion and is required for the development and/or maintenance of the proper glomerular endothelial cell and podocyte architecture (Singh et al., 2007; Wegner et al., 2010). The *cacna1fa* gene controls the voltage-sensitive calcium channel (VSCC) to mediate the entry of calcium ions into and stimulate cells (Figure 8). It also involves various calcium-dependent processes, including muscle contraction, hormone or neurotransmitter release, gene expression, cell movement, and cell division.

## Data availability statement

The datasets presented in this study can be found in online repositories. The names of the repository/repositories and accession number(s) can befound at: https://www.ncbi.nlm.nih.gov/, PRJNA904934, SAMN31858593, SAMN31858594, SAMN31858595, and SAMN31858596.

## Ethics statement

The studies involving human participants were reviewed and approved by Ethics Committee of The Second Xiangya Hospital of Central South University. The patients/participants provided their written informed consent to participate in this study. The animal study was reviewed and approved by Ethics Committee of Hunan Normal University. Written informed consent was obtained from the owners for the participation of their animals in this study. Written informed consent was obtained from the individual(s) for the publication of any potentially identifiable images or data included in this article.

## Author contributions

SY, B-LX, R-sL, and RT Performed the experiment. X-pD, L-xW, G-hZ, and TW analyzed the data. W-jW, F-yC, D-hT, ZD, M-xG, H-pX, YZ, Z-aX, and D-hX written the paper. SY, H-pX, and D-hT designed the experiment. All authors contributed to the article and approved the submitted version.

## Funding

This study was sponsored by the National Natural Science Foundation of China (Grant Nos. 82170308 and 81670938), Xiaoxiang Scholar Distinguished Professor Startup Funding for H-pX.

## Conflict of interest

The authors declare that the research was conducted in the absence of any commercial or financial relationships that could be construed as a potential conflict of interest.

The reviewer JW declared a shared affiliation with the author M-xG to the handling editor at the time of review.

## Publisher’s note

All claims expressed in this article are solely those of the authors and do not necessarily represent those of their affiliated organizations, or those of the publisher, the editors and the reviewers. Any product that may be evaluated in this article, or claim that may be made by its manufacturer, is not guaranteed or endorsed by the publisher.

## Footnotes

^1^http://www.ncbi.nlm.nih.gov/

^2^http://crispor.tefor.net/

## Supplementary material

Supplementary Figure 1 Loss of *cdh23* resulted in curved body. **(A)** WT zebrafish larvae at 96hpf. **(B)** *cdh23*^−/−^. zebrafish larvae at 96hpf. **(C)** Genotyping resulted for siblings and curved body larvae.
