# Supplementary figures and images for: cdh23 affects congenital hearing loss through regulating purine metabolism
Source: Front Mol Neurosci. 2023 Jul 27;16:1079529. doi: 10.3389/fnmol.2023.1079529 (PMC10416109; doi:10.3389/fnmol.2023.1079529)

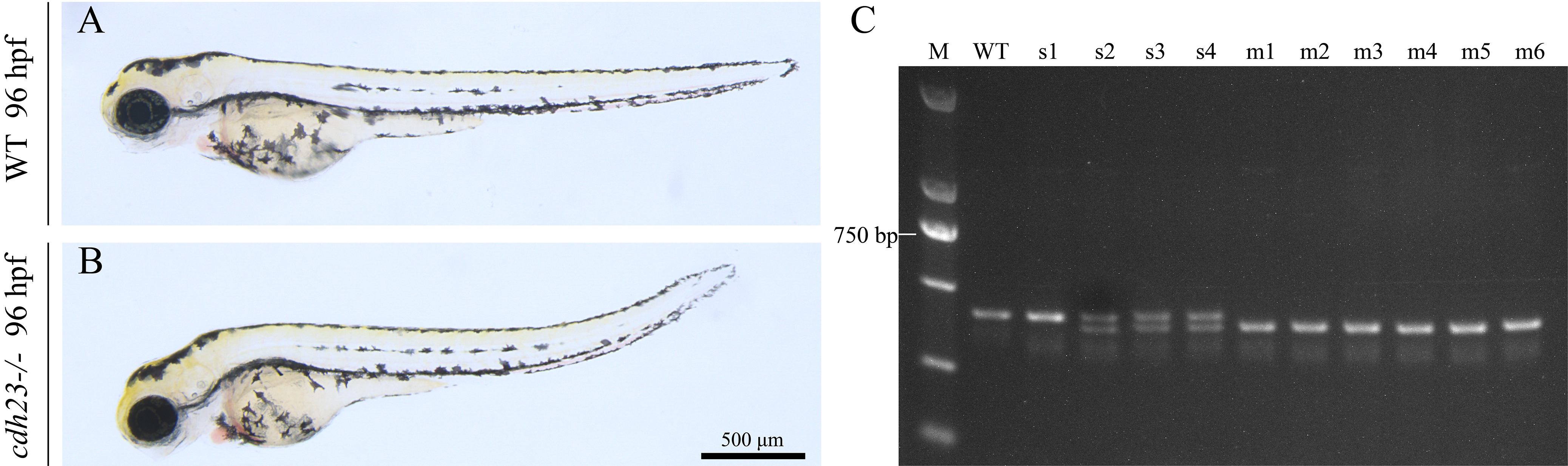

Supplement: Supplementary Figure 1 — Loss of cdh23 resulted in curved body. (A) WT zebrafish larvae at 96hpf. (B) cdh23−/−. zebrafish larvae at 96hpf. (C) Genotyping resulted for siblings and curved body larvae. [file Image_1.jpg]
