# Supplementary material for: cdh23 affects congenital hearing loss through regulating purine metabolism
Source: Front Mol Neurosci. 2023 Jul 27;16:1079529. doi: 10.3389/fnmol.2023.1079529 (PMC10416109; doi:10.3389/fnmol.2023.1079529)

Access for all original data and sequencing raw data

<https://www.jianguoyun.com/c/sd/165654b/18770ea73280bcbc#from=https%3A%2F%2Fwww.jianguoyun.com%2Fc%2Fsd%2F165654b%2F18770ea73280bcbc>


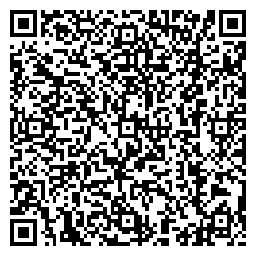

Supplement: Supplementary file 2 [file Data_Sheet_1.docx]
